# Supplementary material for: Force plate methodologies applied to injury profiling and rehabilitation in sport: A scoping review protocol
Source: PLoS One. 2023 Oct 9;18(10):e0292487. doi: 10.1371/journal.pone.0292487 (PMC10561863; doi:10.1371/journal.pone.0292487)
Supplement: S3 File — (DOCX) [file pone.0292487.s003.docx]

| Supplementary file 3. Inclusion/exclusion criteria for literature search. | | | |
| --- | --- | --- | --- |
|  | **Inclusion criteria** | **Exclusion criteria** | **Rationale for these criteria** |
| Population | Athletes competing in individual and team sports. | Paralympic and recreational athletes. | The force plate methodologies used to assess the physical fitness of individuals at high risk of musculoskeletal injuries will be the primary outcome of interest. Thus, only those athletes training and competing regularly will be studied. The heterogeneity of information in paralympic (large number of sport modalities and athlete classification levels) and recreational athletes along with the different injury profiles and demands of sports lead us to exclude studies on these populations. |
| Concept | Force plate methodologies, tests, and metrics applied to assess the injury profiling or rehabilitation process. | Tests using equipment other than force plates. Metrics collected via other technology (e.g., 3-D motion analysis systems). Other force plate methodologies and tests used with non-injury risk reduction purposes. | The aim of this scoping review is to summarise the force plate methodologies which are being implemented to reduce the injury risk in sports. Therefore, only tests and metrics carried out on or extracted from force plates will be included. |
| Context | Primary, secondary and/or tertiary injury prevention. In those studies focused on primary prevention, a measure of the injury occurrence should be reported. | Other study and assessment purposes. Any study that does not report injuries. | Based on the aim of this scoping review, only studies focused on injury profiling and rehabilitation will be included. Other contexts where force plates are used, such as those with physical performance purposes, will be excluded. |
| Publication type | Peer-reviewed original research articles only. | Non-peer-reviewed articles (i.e., pre-print documents), newspapers, opinion pieces, systematic reviews and meta-analysis, editorials, commentaries and letters to the editor, conference proceedings/abstracts, and book chapters. | For reasons of practicality and to avoid duplication of data, it is deemed acceptable to include only studies published in peer-reviewed journals. |
| Language | English and Spanish. | Non- English and Spanish. | According to the researchers’ proficiency, it is deemed acceptable to include only studies published in English or Spanish. |
| Study design | Quantitative study designs, including randomized controlled trials, non-randomized controlled trials, quasi-experimental, before and after studies, prospective and retrospective cohort studies, case-control studies, and cross-sectional studies. | Qualitative studies and literature reviews. | To provide a comprehensive overview of the existing evidence, a broad range of quantitative study designs will be included. |
| Publication date | The date for the last update of the systematic search will be as close as possible to the date of completion of the first draft of the study. | N/A | All articles will be included regardless of the time period. |
